# Pharmacokinetics and pharmacodynamics of fosmidomycin monotherapy and combination therapy with clindamycin in the treatment of multidrug resistant falciparum malaria

**DOI:** 10.1186/1475-2875-6-70

**Published:** 2007-05-25

**Authors:** Kesara Na-Bangchang, Ronnatrai Ruengweerayut, Juntra Karbwang, Anurak Chauemung, David Hutchinson

**Affiliations:** 1 Faculty of Allied Health Sciences, Thammasat University, Pathumthani, Thailand; 2 Mae Sot Hospital, Tak Province, Thailand; 3 UNDP/World Bank/WHO Special Programme for Research and Training in Tropical Diseases (TDR), World Health Organisation, Geneva, Switzerland; 4 Jomaa Pharma GmbH, Schnackenburgallee 116A, 22525 Hamburg, Germany

## Abstract

**Background:**

The study investigated the pharmacokinetics of fosmidomycin when given alone and in combination with clindamycin in patients with acute uncomplicated falciparum malaria.

**Methods:**

A total of 15 and 18 patients with acute uncomplicated *Plasmodium falciparum *malaria who fulfilled the enrollment criteria were recruited from out-patient department of Mae Sot Hospital, Tak Province, Thailand. Patients were treated with monotherapy with fosmidomycin at the dose of 1,200 mg every 8 hours for 7 days (n = 15) or combination therapy with fosmidomycin (900 mg every 12 hours for 7 days) and clindamycin (600 mg every 12 hours for 7 days) (n = 18). Blood samples were taken for pharmacokinetic investigations of clindamycin and/or fosmidomycin and 24-hour urine samples were collected during dosing period. Efficacy assessments included clinical and parasitological evaluation. Safety and tolerability were assessed based on clinical and laboratory investigations.

**Results:**

Both mono- and combination therapy regimens of fosmidomycin were well tolerated with no serious adverse events. Combination therapy with fosmidomycin and clindamycin was proven highly effective with 100% cure rate, whereas cure rate of monotherapy was 22% (28-day follow up). Pharmacokientics of fosmidomycin following mono- and combination therapy were similar except V_z_/F and CL/F, which were significantly smaller in the combination regimen. Plasma concentration-time profiles of both fosmidomycin and clindamycin were best fit with a one-compartment open model with first-order absorption and elimination and with absorption lag time. Steady-state plasma concentrations of fosmidomycin and clindamycin were attained at about the second or third dose. There was no evidence of dose accumulation during multiple dosing. Urinary recovery of fosmidomycin was 18.7 and 20% following mono- and combination therapy, respectively.

**Conclusion:**

Pharmacokinetic dose optimization of fosmidomycin-clindamycin combination therapy with the course of treatment of not longer than three days is required to obtain a regimen which is safe and produced 100% cure for multidrug-resistant *P. falciparum*.

## Background

Malaria is one of the leading causes of morbidity and mortality in the tropics with an annual estimate of 500 million clinical cases and 2 million deaths [1]. The treatment of malaria is becoming increasingly difficult due to *Plasmodium falciparum *strains resistant to commonly used antimalarials. In addition to the high-grade chloroquine-resistant strains found in high frequencies in all endemic areas of the world, resistance to sulphadoxine/pyrimethamine is now common in large parts of Asia and South America and is spreading to Africa as well. Development of drug resistance and in some cases, concerns over safety, highlight the urgent requirement for new antimalarial drugs. Primarily directed towards the treatment of multidrug resistant *P. falciparum*, such drugs should possess novel modes of action while being of proven efficacy and safety.

Fosmidomycin is a phosphonic acid derivative previously investigated as an antibacterial agent and later on has been shown to be an effective malarial blood schizonticide. It acts as a potent inhibitor of 1-deoxy-*D*-xylulose *5*-phosphate (DOXP) reductoisomerase, an essential enzyme of the non-mevalonate pathway and therefore, selectively blocks the biosynthesis of isopentenyl diphosphate and the subsequent development of isoprenoids in *P. falciparum *[2-5]. The mevalonate independent biosynthesis pathway for isoprenoides takes place in special organelles of the parasites, the apicoplasts. These possess, similarly to mitochondria or chloroplasts of green plants, their own genome and can replicate independently of the cell nucleus. Metabolic processes in the apicoplast are similar to those found in bacteria and plants and differ fundamentally from those of animal organisms. Fosmidomycin should, therefore, be very safe in humans in whom isoprenoids are synthesized through a completely different pathway. The drug has been shown *in vitro *and *in vivo *in animal studies to be a potential antimalarial, but the development of recrudescence found in early phase of clinical trial precludes its use as monotherapy [6]. Drug combinations are considered superior to monotherapy with regards to pharmacodynamic synergistic effect and, in addition, the delay of emergence of resistance to the individual components. Clindamycin emerged as a potential partner following the demonstration of *in vitro *and *in vivo *synergistic activity [7]. A subsequent clinical study in Gabon confirmed the effectiveness of this combination in children with asymptomatic malaria to be superior to fosmidomycin and clindamycin, when administered as monotherapy, both in the initial clearance of parasitaemia and in the prevention of recrudescent infections [8,9]. It is not known, however, whether concurrent administration of fosmidomycin and clindamycin would affect the pharmacokinetics of the individual drugs.

The present pharmacokinetic study was part of two phase II clinical trials for dose optimization of fosmidomycin monotherapy (1,200 mg every 8 hours for 7 days) and combination therapy with fosmidomycin (900 mg every 12 hours for 7 days) and clindamycin (600 mg every 12 hours for 7 days) [10]. The study was the first report that characterize pharmacokinetic profiles of fosmidomycin and clindamycin for dose optimization of fosmidomycin mono- and combination therapy with clindamycin in patients with multidrug-resistant falciparum malaria.

## Patients and methods

### Study design

This pharmacokinetic study was part of two open-label, uncontrolled clinical trials to evaluate the clinical efficacy and tolerability of two dosage regimens of fosmidomycin mono- and combination therapy with clindamycin in patients with acute uncomplicated falciparum malaria conducted in Thailand during 2000–2002 [10]. Study protocols were obtained from the Ethics Committees of the Ministry of Public Health of Thailand and the Secretariat Committee for Research Involving Human Subjects (SCRIHS), the World Health Organization, Geneva, Switzerland.

### Patient

A total of 33 patients (15 Thais, 5 Karens, 13 Burmeses) with acute uncomplicated P. falciparum malaria (mono-infection) of either gender, who were admitted to Mae Sot General Hospital, Tak Province, were included in the pharmacokinetic study. Mae Sot is located on the western border of Thailand-Myanmar and is well documented as a multidrug resistance area. Patient inclusion criteria included age between 18–50 years, each with a body weight in excess of 40 kg, attending as outpatients with signs/symptoms of acute uncomplicated malaria and parasitaemia levels between 1,000 and 50,000/μl. Subjects were excluded if they were pregnant or breast-feeding, or had a mixed infection, or significant concomitant disease, or a haemoglobin of < 8.0 g/dl, or white cell count of > 12,000/μl (to exclude severe disease), or had received antimalarial treatment within the previous 28 days.

Pre-treatment investigations consisted of clinical assessments (general medical history, demographic data, drug sensitivity and allergy, significant medical history, previous drug administration, physical examinations, monitoring of vital signs and malaria signs and symptoms), laboratory assessments (thick and thin blood smears for parasite identification/quantification, urine test for chloroquine [11] and sulphonamides [12], routine haematology, serum biochemistry, urinalysis, and stool examination for parasites and ova.

All patients were admitted to the hospitals for seven days and were requested to complete follow-up visits on days 10, 14, 21 and 28 or until recrudescence occurred.

### Drug administration

#### Fosmidomycin monotherapy

Patients were treated with oral doses of fosmidomycin 1,200 mg every 8 hours for 7 days. Fosmidomycin capsules (batch no. 008003) were supplied by Alphamed PHARBIL Arzneimittel GmbH (Germany); each capsule containing 400 mg of fosmidomycin.

#### Fosmidomycin combination therapy

Patients were treated with oral fosmidomycin concurrently with clindamycin. Fosmidomycin was given at the dose of 900 mg every 12 hours for 7 days. Fosmidomycin capsules (batch no. FK 150–02007) were supplied by Alphamed PHARBIL Arzneimittel GmbH; each capsule containing 150 mg of fosmidomycin. Clindamycin was given at the dose of 600 mg every 12 hours for 7 days. Clindamycin film coated tablets (batch number 16843-007) were supplied by Alphamed PHARBIL Arzneimittel GmbH; each tablet containing 150 mg of clindamycin.

The patients fasted over night (8–10 hr) and emptied their bladders immediately before drug administration. The drugs were administered orally with 250 ml of water and standard hospital meal (20–25% fat content) under supervision of the assigned study staff. After ingestion of drug tablets, patients were observed for 1 hr to ensure retention of the drug. Patients who vomited any oral dose of fosmidomycin or clindamycin were excluded from pharmacokinetic data analysis. Meal was provided at 4 hr post-dosing. Juices and water were freely available during hospitalisation. Except for drugs described in the exclusion criteria, all drugs necessary for the welfare of the patients were allowed during the study period.

### Blood and urine collection

A total of 15 blood samples (3 ml each) for pharmacokinetic investigations of fosmidomycin monotherapy were collected from patients during days 0–7 of drug administration as follows: at 0 (before first dose), 1, 2, 4, 8, 16, 24 (day 1), 48 (day 2), 72 (day 3), 96 (day 4), 120 (day 5), 144 (day 6), 168 (day 7), 192 and 216 h. For combination therapy regimen, a total of 12 blood samples (5 ml each) for pharmacokinetic investigations of fosmidomycin and clindamycin were obtained from patients during days 0–7 of dosing as follows: at 0 (before first dose), 24 (day 1), 48 (day 2), 156 (day 7), 157, 158, 160, 162, 164, 168, 174 and 180 hr from the commencement of dosing. Samples collected at the time of drug administration were taken prior to the dose. Blood samples were centrifuged (3,000 g, 15 min), and plasma samples were stored at -20°C until analysis.

Twenty-four hours urine samples were collected from all patients over 7–10 days from the commencement of dosing. The volume of each urine collection was measured and aliquots of 10 ml for the specified time periods were collected into a 10 ml-polypropylene tube and stored at -70°C until analysis.

### Efficacy assessment

Efficacy assessments included clinical and parasitological evaluation. Clinical signs/symptoms of malaria including body temperature were monitored on days 0, 1, 2, 3, 4, 5, 6, 7, 10, 14, 21 and 28. Finger-prick blood smears were examined at 6 hourly intervals until negative at 3 consecutive times, then daily during hospitalisation, on days 10, 14, 21 and 28. The films were stained with Giemsa stain and parasite counts were determined by counting the number of asexual parasites per 1,000 red blood cells on a thin film or per 200 white blood cells on a thick film. Analysis of the response to treatment was adapted from the World Health Organization [13]. Efficacy was assessed using the following parameters: (i) 28 day cure rate: the proportion of patients with clearance of asexual parasites within 7 days of treatment initiation without subsequent recrudescence during 28 day follow-up); (ii) 7 day cure rate: the proportion of patients with clearance of asexual parasites within 7 days of treatment initiation; (iii) parasite clearance time (PCT): defined as time from first dose to continued clearance of asexual parasite forms which remained for at least a further 48 h; and (iv) fever clearance time (FCT): time from first dose until the first time body temperature fell below 37.5°C and remained below 37.5°C for at least a further 48 hr.

### Safety and tolerability assessment

Safety and tolerability of fosmidomycin monotherapy and combination therapy with clindamycin were assessed based on clinical findings or abnormal laboratory (haematology and serum biochemistry) tests that first occurred or increased in intensity within 7 days of treatment initiation, and during follow up period on day 14, 21 and 28, in accord with the Common Toxicity Criteria CTC grade [15].

### Drug analysis

Determination of plasma and urine concentrations of fosmidomycin and clindamycin were performed within six months of sample collection. Stability of these two drugs was found to be good under storage condition (-20°C) for a minimum of twelve months [15,16]. Concentrations of fosmidomycin in plasma and urine samples were determined by bioassay system based on agar disk diffusion technique using *Enterobacter cloacae *ATCC 23355 as the test organism [15]. The test medium was prepared from Trypticase soy broth (BBL Microbiology Systems, MDs, USA). The overnight culture in Trypticase soy broth was inoculated into the medium to give a final concentration of 0.5% (v/v). Ten millilitres of the incubated agar medium was poured into a 9-cm Petri dish and incubated at 37°C for 18 to 20 hours. The zones of inhibition were measured and concentrations were estimated from the log-concentration-response curve. The precision of the assay method based on within-day repeatability and reproducibility (day-to-day variation) was below 5% (% coefficient of variation: %C.V.). Good accuracy was also observed for both the intra-day or inter-day assays. Limit of quantification was 1 ng using 40 μl plasma or 7.5 μl urine sample. The method was found to be comparable to capillary electrophoresis method (HPCE).

Concentrations of clindamycin in plasma samples were determined by high performance liquid chromatography as described previously [16]. In brief, clindamycin was separated from the internal standard (phenobarbital) on a Luna C18 column (250 – 4.6 mm, 5 μm particle size: Phenomenex™, USA), with retention times of 5.6 and 14.2 min, respectively. Ultraviolet detection was set at 210 nm. The mobile phase consisted of a solution of 0.02 M disodiumhydrogenphosphate (pH 2.8) and acetonitrile (76:24 v/v), running through the column at a flow rate of 1.0 ml/min. The chromatographic analysis was operated at 25°C. Sample preparation (1 mL plasma) was done by a single step liquid-liquid extraction with water saturated ethylacetate. The precision of the method based on within-day repeatability and reproducibility (day-to-day variation) was below 15% (% coefficient of variations: %C.V.) Good accuracy was observed for both the intra-day or inter-day assays, as indicated by the minimal deviation of mean values found with measured samples from that of the theoretical values (below ± 15%). Limit of quantification was accepted as 0.07 μg using 1 ml plasma sample. The mean recoveries for clindamycin and the internal standard were greater than 95%.

### Pharmacokinetics

The non-compartmental pharmacokinetic analysis was applied to determine the pharmacokinetic parameters of fosmidomycin and clindamycin following a single initial (fosmidomycin monotherapy) or last oral dose (fosmidomycin combination therapy) of fosmindiomycin and clindamycin following oral administration. C_max _was the maximum plasma concentrations and t_max _was the time of the maximum measured plasma concentrations following a single initial or last dose. C_min-obs _was the observed trough concentration. The area under the concentration-time curve from time zero to the last sampling point that yielded a quantifiable concentration (AUC_0, t_) was calculated with the log-linear trapezoidal method. At least the last three sampling points were used to determine the first-order constant associated with the terminal portion of the curve (λ_z_). Terminal phase elimination half-life (t_1/2z_) was derived as t_1/2z _= ln(2)/λ_z_. The AUC from time zero to infinity (AUC_0, ∞_) was calculated as AUC_0-t _+ Ct/λ_z_, where C_t _stands for the last measurable concentration. Total clearance (CL/F) was calculated from Dose/AUC_0,∞ _and apparent volume of distribution (V_z_/F) was calculated as CL/F/λ_z_, where F is bioavailability. Assuming bioavailability (F) of 1 for both fosmidomycin and clindamycin, maximum plasma concentration at steady-state (C_max-ss_), minimum plasma concentration at steady-state (C_min-ss_) and average plasma concentration at steady-state (C_ave-ss_) following fast release oral formulation were estimated from the relationships:

Cmax⁡−ss=DoseVz/F(1−e−λ.τ)
 MathType@MTEF@5@5@+=feaafiart1ev1aaatCvAUfKttLearuWrP9MDH5MBPbIqV92AaeXatLxBI9gBaebbnrfifHhDYfgasaacH8akY=wiFfYdH8Gipec8Eeeu0xXdbba9frFj0=OqFfea0dXdd9vqai=hGuQ8kuc9pgc9s8qqaq=dirpe0xb9q8qiLsFr0=vr0=vr0dc8meaabaqaciaacaGaaeqabaqabeGadaaakeaaieaacqWFdbWqdaWgaaWcbaGagiyBa0MaeiyyaeMaeiiEaGNaeyOeI0Iae83CamNae83Camhabeaakiabg2da9maalaaabaGae8hraqKae83Ba8Mae83CamNae8xzaugabaGae8Nvay1aaSbaaSqaaiab=Pha6bqabaGccqGGVaWlcqWFgbGrcqGGOaakcqaIXaqmcqGHsislcqWFLbqzdaahaaWcbeqaaiabgkHiTGGaciab+T7aSjab+5caUiab+r8a0baakiabcMcaPaaaaaa@4B51@

C_min-ss _= C_max-ss_.e^-λ.τ^

Cave-ss=DoseCL/F.τ
 MathType@MTEF@5@5@+=feaafiart1ev1aaatCvAUfKttLearuWrP9MDH5MBPbIqV92AaeXatLxBI9gBaebbnrfifHhDYfgasaacH8akY=wiFfYdH8Gipec8Eeeu0xXdbba9frFj0=OqFfea0dXdd9vqai=hGuQ8kuc9pgc9s8qqaq=dirpe0xb9q8qiLsFr0=vr0=vr0dc8meaabaqaciaacaGaaeqabaqabeGadaaakeaacqqGdbWqdaWgaaWcbaGaeeyyaeMaeeODayNaeeyzauMaeeyla0Iaee4CamNaee4Camhabeaakiabg2da9maalaaabaGaeeiraqKaee4Ba8Maee4CamNaeeyzaugabaGaee4qamKaeeitaWKaee4la8IaeeOrayKaeeOla4ccciGae8hXdqhaaaaa@42D2@

Where τ is dosing interval (hr).

Fluctuation between C_max-ss _and C_min-ss _was calculated from the ratio between the two parameters.

In order to simulated plasma concentration-time profiles of fosmidomycin and clindamycin when given as mono- and combination therapy, one-compartment open model with first-order absorption and elimination with absorption lag time were applied using ADAPT II, release 4.0 [17].

### Statistical analysis

Descriptive statistics were calculated for pharmacokinetic variables. Geometric means were determined for baseline parasite counts. All pharmacokinetic parameters were presented as median (95% C.I.). Comparison of baseline data (demographic, clinical, laboratory) with the data obtained during follow up was performed using the Wilcoxon-Signed Rank test. Comparison of the pharmacokinetic parameters between the two groups was performed using the Mann-Whitney U test. The level of statistical significance was set at α = 0.05 for all tests.

## Results

Demographics, admission clinical and laboratory data of the groups of patients are presented in Table 1. Most patients had baseline laboratory parameters outside the normal ranges; major parameters are presented in the table. Most are common in patients with acute falciparum malaria and all were mild or moderate in severity. A total of 15 and 18 patients received treatment with fosmidomycin monotherapy and combination therapy with clindamycin, respectively. Two patients (No 305, 314) who received fosmidomycin monotherapy had successful treatment, while the other nine cases developed recrudescence and four lost to follow up during 28 day period. Seven- and 28-day cure rate following monotherapy *vs *combination therapy were 100 (10/10) and 22 (2/9)% *vs *100 (12/12) and 100 (12/12)%, respectively. The corresponding values [median (range) values] for FCTs and PCTs for mono- *vs *combination therapy were 56 (24–104) and 56 (8–80) hr *vs *40 (24–72) and 40 (16–80) hr, respectively. Concomitant medication included paracetamol, dimenhydinate, diphenhydramine and chlorpheniramime.

**Table 1:** Demographics, admission clinical and laboratory data of patients with acute uncomplicated falciparum malaria who were treated with fosmidomycin monotherapy and combination therapy with clindamycin. Data are presented as median (95% C.I.) or number of patients and percentage (n, %) values.

**Characteristics**	**Fosmidomycin monotherapy (N = 15)**	**Fosmidomycin + Clindamycin (N = 18)**
Age (yr)	36 (19–45)	29 (18–48)
Body weight (kg)	55 (42–67)	56 (40–75)
Male/Female	11/4	13/5
Ethnic:		
Thai	5	10
Karen	2	3
Burmese	8	5
Body temperature (°C)	38.3 (36.0–40.0)	38.0 (37.3–39.9)
Baseline asexual form parasitaemia (/μl)^a^	11,747 (1,141–173,880)	30,600 (944–67,640)
Haemoglobin (mg/dl)	12.4 (8.0–15.5)	12.6 (8.1–15.0)
Haematocrit (%)	38.6 (26.5–44.8)	39.8 (25.5–45.7)
WBC (x10^9^/l)	5.8 (3.5–8.7)	6.0 (3.0–8.9)
Platelet count (x10^9^/l)	78 (22–230)	80 (21–225)
Total bilirubin (mg/dl)	1.5 (0.3–2.5)	1.3 (0.1–2.6)
ALT (IU/l)	25 (10–98)	24 (8–103)
Creatinine (mg/dl)	1.2 (0.9–1.8)	1.1 (0.7–1.9)
BUN (mg/dl)	13 (9–40)	14 (7–43)
Total protein (mg/dl)	6.2 (5.3–8.1)	6.0 (5.0–8.0)
Albumin (g/dl)	3.8 (3.0–4.6)	3.6 (2.9–4.5)
Glucose (mg/dl)	118 (80–198)	110 (79–205)

^a^geometric mean (range)

### Pharmacokinetics and urinary excretion

**Figure 1:**
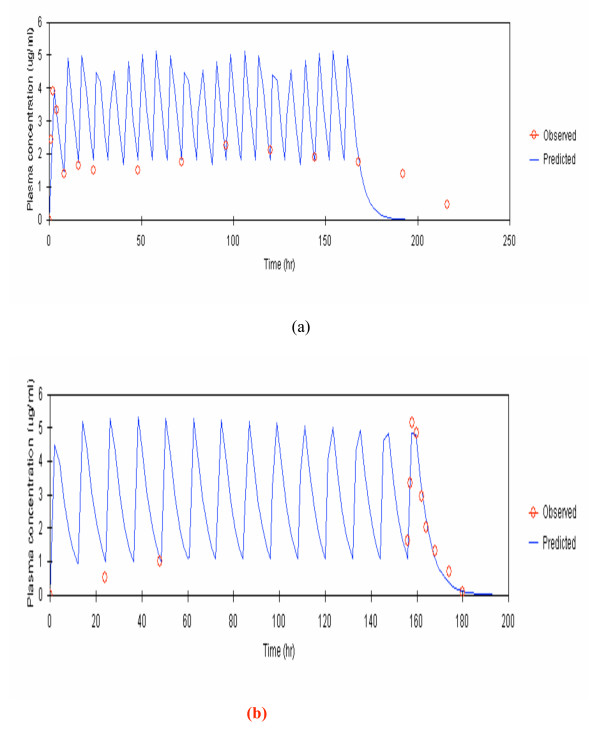
Observed and predicted median plasma concentrations of fosmidomycin (by one-compartment open model with first-order absorption and elimination with absorption lag time) given as (a) monotherapy (1,200 mg every 8 hours for 7 days), and (b) combination therapy with clindamycin (900 mg every 12 hours for 7 days).

**Figure 2:**
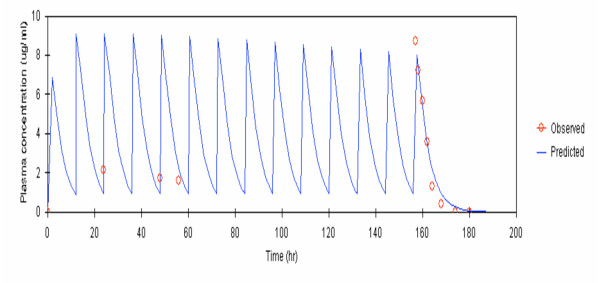
Observed and predicted median plasma concentration of clindamycin (by one compartment open model with first-order absorption and elimination with absorption lag time) given as combination therapy with fosmidomycin (600 mg every 12 hours for 7 days).

Observed and model-predicted (one-compartment model with first-order absorption and elimination with absorption lag time) median plasma concentrations of fosmidomycin and clindamycin following monotherapy and combination therapy are shown in Figures 1(a,b) and 2.

The pharmacokinetics of fosmidomycin given as monotherapy and combination therapy including pharmacokinetics of clindamycin given as combination therapy with fosmidomycin in individual patients analysed by non-compartmental pharmacokinetic analysis approach are summarized in Table 2. Variability as expressed as % C.V. for all parameters varied between 7.7 and 40.5%. V_z_/F and CL/F were significantly smaller following combination therapy; other pharmacokinetic parameters were comparable between the two treatment regimens.

**Table 2:** Pharmacokinetics of fosmidomycin and clindamycin analysed by non-compartmental model in patients with acute uncomplicated falciparum malaria. Data are presented as median (95% C.I.) values.

**Pharmacokinetic parameters**	**Fosmidomycin monotherapy (N = 15)**	**Fosmidomycin + Clindamycin (N = 18)**	
		
		**Fosmidomycin**	**Clindamycin**
C_max _(μg/ml)	4.58 (2.32–6.77)	5.86 (2.79–12.52)	7.34 (4.88–12.73)
t_max _(hr)	2 (1–4)	2 (1–4)	1 (1–2)
C_trough-obs _(μg/ml)	1.90 (0.92–2.99)	0.40 (0.0–2.01)	1.32 (0.39–3.42)
C_min-ave _(μg/ml)	1.47 (0.06–9.96)	1.06 (0.20–2.98)	0.17 (0.01–0.78)
C_max-ave _(μg/ml)	7.22 (1.52–15.84)	8.67 (4.21–14.23)	6.63 (3.96–9.52)
C_ss-ave _(μg/ml)	3.74 (3.18–12.52)	2.93 (1.97–6.98)	1.91 (1.20–3.06)
Fluctuation	5.1 (1.6–47.6)	10.5 (3.6–40.3)	1.1 (0.7–1.6)
AUC_0, t _(μg.hr/ml)	22.91 (12.65–33.32)	35.00 (20.41–78.90)	31.80 (15.84–370.67)
AUC_0_,((μg.hr/ml)	29.7 (25.06–100.16)	39.13 (24.29–85.55)	31.91 (19.28–50.49)
λ _Z _(/hr	0.201 (0.06–0.48)	0.196 (0.107–0.308)	0.272 (0.156–0.464)
T_1/2z _(/hr)	3.4 (1.4–11.8)	3.5 (2.2–6.5)	2.6 (1.5–4.4)
V_Z_/F (l)	211.40 (96.84–823.04)	116.21 (69.94–267.45)^a^	67.50 (46.56–135-87)
CL/F (l/hr)	40.08 (11.97–47.18)	25.94 (10.74–38.13)^b^	18.77 (11.77–30.12)

a = Statistically significant difference with monotherapy with p-value = 0.004

b = Statistically significant difference with monotherapy with p-value < 0.0001

Table 3 summarizes pharmacokinetics of fosmidomycin and clindamycin analysed by one-compartment open model with first order absorption and elimination with absorption lag time. Steady-state plasma levels of fosmidomycin were reached at about 16–24 hr (third dose) following both regimens, while steady-state plasma levels of clindamycin were reached at earlier time of 12–24 hr (second dose).

**Table 3:** Pharmacokinetics of fosmidomycin and clindamycin analysed by one-compartment model with first order absorption and elimination with absorption lag-time. Data are presented as median (%C.V) values.

**Pharmacokinetic parameters**	**Fosmidomycin monotherapy (N = 15)**	**Fosmidomycin + Clindamycin (N = 18)**	
		
		**Fosmidomycin**	**Clindamycin**
C_max _(μg/ml)	3.95 (12.8)	4.06 (6.8)	5.98 (5.1)
t_max _(hr)	2.24 (28.1)	2.46 (11.6)	1.14 (2.4)
K_01 _(/hr)	1.27 (104.3)	1.17 (2.5)	3.99 (111.3)
K_10 _(/hr)	0.20 (24.7)	0.18 (12.5)	0.22(0.1)
t_lag _(hr)	0.5 (83.3)	0.6 (15.4)	0.38(17.6)
AUC_0_, (μg.hr/ml)	27.67 (7.6)	35.59 (4.1)	31.9(75.9)
t_1/2a _(hr)	0.5 (104.0)	0.6 (39.9)	0.17(4.8)
t_1/2e _(hr)	3.4 (24.7)	3.8 (13.0)	3.11(0.7)
V_z_/F (l)	214.00 (27.4)	138.00 (14.9)	84 (21.0)
CL/F (l/hr)	433.66 (7.7)	252.85(4.1)	18.79(44.0)

k_01 _= absorption rate constant; k_10 _= elimination rate constant; t_lag= _absorption lag time; t_1/2a _= absorption half-life; t_1/2e_= elimination half-life

Urinary concentrations of fosmidomycin were determined during the dosing period of 7–10 days and results showed that percentage of total amount of fosmidomycin excreted during 96–120 hr were markedly high following both mono- and combination therapy of fosmidomycin. There was no significant difference in urinary recoveries of fosmidomycin (expressed as percent of dose administered) following monotherapy and combination therapy [median (95% C.I.): 18.7 (6.5–42.1) *vs *20.0 (6.9–43.3)%].

### Relationship between plasma drug concentration and therapeutic outcome

C_min-ss _of fosmidomycin in two patients with successful treatment on day 28 following fosmidomycin monotherapy were 2.16 and 1.24 μg/ml, while the levels in 9 patients who developed recrudescence were 1.19, 1.42, 2.79, 0.67, 0.27, 4.57, 1.95, 0.06 and 1.47 μg/ml. In the group treated with fosmidomycin-clindamycin combination therapy, all were cured despite relatively low C_min-ss _of 0.20 μg/ml. The pharmacokinetics of fosmidomycin in patients who had successful or treatment failure following fosmidomycin monotherapy appeared to be similar.

### Adverse effects

Fosmidomycin mono- and combination therapy with clindamycin were well tolerated with no serious adverse events, deaths or withdrawals due to adverse events. No neutropaenia or drop in haemoglobin were observed as previously reported in children [9]. Following fosmidomycin monotherapy, four (33.3%), three (25.0%), two (12.5%) and three (25.0%) cases experienced headache, epitaxis, vertigo and diarrhoea, respectively. Following combination therapy with clindamycin, six (33.3%), one (5.5%), one (5.5%), six (33.3%), three (16.7%) and one (5.5%) cases experienced diarrhoea, dizziness, epitaxis, headache, anorexiamacular rash, and myalgia respectively. In addition, elevated ALT and hypokalaemia were found in four (22.2%) and one (5.5%) cases, respectively. All adverse events were categorized as mild or moderate in severity. Significant within-subject differences over time (days 0, 2, 4, 7, 28 or withdrawal) were found for all laboratory parameters measured, which reflects changes normally encountered during a malarial infection.

## Discussion

A bioassay based on disk diffusion technique was applied for determination of fosmidomycin in plasma and urine as the drug is the only bioactive compound. The analysis of plasma concentrations of clindamycin was performed by HPLC since bioassay lacks ability to discriminate between clindamycin and its active metabolites (*N*-desmethyl clindamycin and clindamycin sulphoxide) [18-20]. Fosmidomycin and clindamycin were well absorbed orally, but bioavailability of fosmidomycin may not be complete which may be due to its high water solubility property [21]. The bioavailability of fosmidomycin and clindamycin have been reported to be 30 and 72%, respectively [18,19,22]. Pharmacokinetics of fosmidomycin and clindamycin following multiple oral dosing either as mono- or combination therapy in patients with acute uncomplicated falciparum malaria observed in this study are comparable with those reported in healthy subjects following a single dose of 500 mg or multiple doses of 250 or 500 mg given every 6 hours [21,23,24]. In single dose studies, mean peak plasma concentrations of 2.45 μg/ml were attained following doses of 500 mg. In repeated dose studies in which fosmidomycin was administered every 6 hours for periods of 5 to 7 days in doses of 250, 500 mg and 1 g, steady-state concentrations of 2.45, 4.01 and 5.0 μg/ml, respectively were achieved after 24 hr. Plasma concentration-time profiles of both drugs were best fitted with a one-compartment open model with first-order absorption and elimination with absorption lag time. It is noted for a marked variability in fluctuation of C_max-ss _and C_min-ss _of fosmidomycin and clindamycin following multiple dosing regimens. The trough/peak plasma concentrations after both dosage regimens increased up to the second or third dose and remained at steady-state thereafter. Fosmidomycin was not metabolised and excreted unchanged in urine as a bioactive substance. The urinary recovery expressed as percent of the administered doses were relatively low (18 and 20% following mono- and combination therapy) comparing to those reported in healthy subjects (26%) [24,25]. This extent of urinary excretion supports the low bioavailability of the drug after oral dose administration. Elimination of both fosmidomycin and clindamycin were not affected by dosing, which suggests no accumulation and dose linearity. This was supported by the consistency of C_max-ss _and C_av-ss _values, the unchanged elimination half-lives after the first and last dose, and the constant urinary recovery during 0–7 days of dosing. In a previous study, saturated absorption of fosmidomycin after multiple dosing was suggested as there were a progressive decline in total urinary recovery from 35.9 to 22.4% during multiple dosing [21].

The non-compartment pharmacokinetic analysis of fosmidomycin monotherapy was investigated during the acute phase of malaria infection on the first day whereas its kinetics following combination therapy with clindamycin was investigated following the last dose on day 7. Data suggest that malaria infection may not have significant influence on the pharmacokinetics of both fosmidomycin and clindamycin. Although amount of fosmidomycin excreted in urine appears to be relatively low in patients with malaria compared with healthy subjects, overall pharmacokinetic profiles were similar. The pharmacokinetics of fosmidomycin when given alone or with clindamycin were generally comparable except for the significant paralleled reduction of V_z_/F and CL/F when clindamycin doses were added, while t_1/2z _remained unchanged. It is not clear whether these changes are contributed mainly by malaria infection or pharmacokinetic interaction with clindamycin or both. Reduction of renal clearance of fosmidomycin when clindamycin was given concurrently may account partly for this pharmacokinetic difference. Fosmidomycin is only eliminated by renal clearance while clindamycin is metabolised by CYP3A in liver to clindamycin sulphoxide and *N*-desmethyl clindamycin [25]. Plasma protein binding of fosmidomycin in human is about 1% but clindamycin binds extensively about 90% to plasma protein, mainly to α_1_-acid glycoprotein [19-23]. Therefore, the interaction if actually occurred, cannot be explained at the level of hepatic metabolism or plasma protein binding displacement.

Fosmidomycin monotherapy was shown safe and effective blood schizonticide in the initial clearance of asexual parasitaemia. However, the high rate of recrudescence precludes its use in monotherapy [6,10] Monotherapy with fosmidomycin was investigated in a pilot study for 1,200 mg every eight hours for seven days with cure rate of 100% (10/10). When the duration was reduced to five, four and three days, cure rates decreased to 89, 88 and 60%, respectively. These observations signify the importance of optimal fosmidomycin dose regimens in the treatment of malaria. In the present study, the cure rate of 22% was observed with monotherapy with fosmidomycin at a dose of 1,200 mg administered orally every eight hours for seven days in a total of 15 Thai patients [10]. The cure rate with the same dose regimen was, however, 100% in Gambian patients [10]. MIC (minimum inhibitory concentration) of fosmidomycin for the treatment of multidrug resistant falciparum malaria remains to be determined but is expected to be higher than that in Gambian isolates. The IC_50 _value for recombinant *P. falciparum *DOXP reductoisomerase is approximately 40 nM and varied from 300 to 1,200 nM in *P. falciparum in vitro *without obvious cross-resistance with other antimalarials [7,26]. Plasma concentrations in two patients with successful treatment following monotherapy were as high as 1.24 and 2.1 μg/ml, which are considered adequate for radical cure. It was noted that some patients among the 10 cases had recrudescence despite plasma concentrations markedly greater than these levels (4.52 μg/ml). This suggests intrinsically low sensitivity of some parasite strains in Thailand to fosmidomycin and that monotherapy of the drug may not be effective for treatment of multidrug resistant falciparum malaria. When fosmidomycin was given with clindamycin (900 mg fosmidomycin in combination with clindamycin at 600 mg administered orally every 12 hours for 7 days) on the other hand, radical cure was achieved in all patients even with the trough concentrations of as low as 0.2 μg/ml. This could be explained by pharmacodynamic synergistic interaction between fosmidomycin and clindamycin on parasite enzyme DOXP reductoisomerase. Clindamycin targets the prokaryote-like ribosomes of the apicoplast and by this means inhibits self-replication of the organelle [27,28]. As a consequence of this mechanism, the drug displays a typical delayed kill kinetic effect, the growth of parasites being unaffected until the second replication after drug exposure. Under such conditions, the *in vitro *growth of *P. falciparum *is inhibited with an IC_50 _and IC_90 _of approximately 25 and 50 nM, respectively. As a consequence of its high activity but slow onset of activity, clindamycin is recommended for the treatment of asymptomatic malaria or in combination with other antimalarials. It has been suggested that time-dependent antimicrobial agents should remain above the MIC of pathogens for at least three malaria cycle of seven days. Unlike fosmidomycin, recent studies have suggested that clindamycin displays concentration-dependent bacteriocidal activity [17,29] while exhibiting an *in vitro *post antibiotic effect (PAE) for certain period. *In vivo*, the PAE is generally longer and due to effects such as post antibiotic leukocyte enhancement and post-antibiotic sub-MIC effect. Frequent dosing interval of clindamycin regimen is therefore not necessary. The recommended oral dosing of clindamycin for bacterial infection was 600 mg every 6 hr or 900 mg every 8 hr. However, it was later shown that the pharmacokinetics and concentration-time profiles were comparable with the dosing regimen 1,200 mg every 12 hr which is more practical for clinical application. This may explain the sustained antimalarial efficacy when clindamycin was used with fosmidomycin despite low minimum plasma concentrations of fosmidomycin at steady-state. Whether the antimalarial activity of clindamycin bioactive metabolites should also be considered when performing a pharmacokinetic/pharmacodynamic evaluation has not yet been elucidated. In human, the formation of *N*-desmethyl clindamycin and its excretion in urine and faece have been confirmed [25] but the metabolite could not be detected in the plasma of patients [30]. The currently suggested dose regimens are based on parent compound concentrations only and the presence of metabolite activity (if any) would only amplify the positive therapeutic outcome. Further study is required for dose optimization of the appropriate ratio of fosmidomycin and clindamycin combination including dosing interval with shorter course of at least 3 days that is safe and effective (produces 100% cure) for treatment of multidrug resistant falciparum.

## Abbreviations

AUC Area under the plasma concentration-time curve

C_min-obs _Observed trough concentration

C_max _Maximum plasma concentration

C_max-ss _Maximum plasma concentration at steady-state

C_ave-ss _Average plasma concentration at steady-state

CI Confidence interval

CL Total clearance

CV Coefficient of variation

F Bioavailability

FCT Fever clearance time

HPCE High performance capillary electrophoresis

HPLC High performance liquid chromatography

IC_50 _Fifty percent inhibitory concentration

MIC Minimum inhibitory concentration

PAE Post antibiotic effect

PCT Parasite clearance time

t_max _Time to maximum plasma concentration

V_z _Apparent volume of distribution

## Authors' contributions

I Kesara Na-Bangchang was overall study supervisor and analyst of pharmacokinetic data including co-author for the manuscript. R Ruenweerayut was the site investigator and overall medical coordinator. J Karbwang and D Huchington reviewed the study protocol and manuscript and and provided technical support. A Chauemung was responsible for the analysis of pharmacokinetic samples (determination of fosmidomycin and clindamycin in plasma and urine).

## Authors' contributions

KN participated in the design of the study, performed pharmacokinetic and statistical analysis, as well as drafting the manuscript. RR was responsible for the conduct of clinical part of the study (patient's recruitment, treatment and data and sample collection). AC carried out the analysis of fosmidomycin and clindamycin in plasma and urine samples. JK and DH conceived of the study, and participated in its design and coordination. All authors read and approved the final manuscript.

## References

[B1] World Health Organization (1997). World malaria situation in 1994. Wkly Epidemiol Rec.

[B2] Jomaa H, Wiesner J, Sanderbrand S, Altincicek B, Weidemeyer C, Hintz M, Turbachova I, Eberl M, Zeidler J, Lichtenthalter HK, Soldati D, Beck E (1999). Inhibitors of the nonmevalonate pathway of isoprenoid biosynthesis as antimalarial drugs. Science.

[B3] Kuzuyama T, Shizimu T, Takashi S, Seto H (1998). Fosmidomycin, a specific inhibitor of 1-deoxy-D-xylulose 5-phosphate reductoisomerase in the nonmevalonate pathway of isoprenoid biosynthesis. Tetrahaedron Lett.

[B4] Rohmer M, Knani M, Simonin P, Sutter B, Sahm H (1993). Isoprenoid biosynthesis in bacteria: a novel pathway for the early steps leading to isopentenyl diphosphate. Biochem J.

[B5] Zeidler J, Schwender J, Mûller C, Wiesner J, Weidemeyer C, Beck E, Jomaa H, Lichtenthaler HK (1993). Inhibition of the non-mevalonate 1-deoxy-D-xylulose-5-phosphate pathway of plant isoprenoid biosynthesis by fosmidomycin. Z Naturforsch.

[B6] Missinou MA, Borrmann S, Schindier A, Issifou S, Adegnika AA, Matsiegui PB, Binder B, Lell B, Wiesner J, Baranek T, Jomaa H, Kremsner PG (2002). Lancet.

[B7] Wiesner J, Henschker D, Hutchinson DB, Beck E, Jomaa H (2002). *In vitro *and *in vivo *synergy of fosmidomycin, a novel antimalarial drug, with clindamycin. Antimicrob Agents Chemother.

[B8] Borrmann S, Adegnika AA, Matsiegui PB, Issifou S, Scindier A, Mawilli-Mboumba DP, Baranek T, Jomaa H, Kremsner PG (2004). Fosmidomycin-clindamycin for *Plasmodium falciparum *infections in African children. JID.

[B9] Borrmann S, Lundgren I, Oyakhirome S, Impouma B, Matsiegui PB, Adegnika AA, Issifou S, Kun JF, Hutchinson D, Wiesner J, Jomaa H, Kremsner PG (2006). Fosmidomycin plus clindamycin for treatment of pediatric patients aged 1 to 14 years with *Plasmodium falciparum *malaria. Antimicrob Agents Chemother.

[B10] Lell B, Ruangweerayut R, Wiesner J, Missinou MA, Schindler A, Baranek T, Hintz M, Hutchinson DB, Jomaa H, Kremsner PG (2003). Fosmidomycin, a novel chemotherapeutic agent for malaria. Antimicrob Agents Chemother.

[B11] Lelijveld J, Kortmann H (1970). The eosin colour test of Dill and Glazko: a simple field test to detect chloroquine in urine. Bull World Health Organ.

[B12] De Almeida-Filho  J, de Souza JM (1983). A simple urine test for sulfonamides. Bull World Health Organ.

[B13] World Health Organization, Division of Control of Tropical Diseases (1996). Assessment of therapeutic efficacy of antimalarial drugs for uncomplicated malaria in areas with intense transmission.

[B14] Cancer Therapy Evaluation Programes (1998).

[B15] Cheoymang A, Hudchinton D, Kioy D, Na-Bangchang K (2006). Bioassay for determination of fosmidomycin in plasma and urine: application for pharmacokinetic dose optimization. J Microb Methods.

[B16] Na-Bangchang K, Banmairuroi V, Kamanikom B, Kioy D (2006). An alternative high-performance liquid chromatographic method for determination of clindamycin in plasma. Southeast Asian J Trop Med Public Health.

[B17] d' Argenio  DZ, Schumitzky A (2006). Pharmacokinetic/pharmacodynamic system analysis software.

[B18] De Hann  RM, Metzler CM, Schllenberg D, VandenBosch GW (1973). Pharmacokinetic studies of clindamycin phosphate. J Clin Pharmacol.

[B19] Metzler CM, De Hann R, Schellenberg D, VandenBosch WD (1973). Clindamycin dose-bioavailability relationships. Pharm Sci.

[B20] Wagner JG, Novak GE, Patel NC, Chidester CG, Lummis WL (1968). Absorption, excretion and half-life of clindamycin in normal adult males. Am J Med Sci.

[B21] Kuemmerle HP, Murakawa T, Sakamoto H, Sato N, Konishi T, De Santis  F (1985). Fosmidomycin, a new phosphonic acid antibiotic. Part II: 1. Human pharmacokinetics. 2 Preliminary early phase IIa clinical studies. Int J Clin Pharmacol Ther Toxicol.

[B22] Kuemmerle HP, Muragawa T, De Santis F (1987). Pharmacokinetic evaluation of fosmidomycin, a new phosphonic acid antibiotic. Chemotherapia.

[B23] Murakawa T, Sakamoto H, Fukada S, Konishi T, Nishida M (1982). Pharmacokinetics of fosmidomycin, a new phosphonic acid antibioti. Antimicrob Agents Chemother.

[B24] Wynalda MA, Hutzler JM, Koets MD, Podoll T, Wienkers LC (2003). *In vitro *metabolism of clindamycin in human liver and intestinal microsomes. Drug metabol Dispos.

[B25] Neu HC, Kamimura T (1982). Synergy of fosmidomycin (FR-31564) and other antimicrobial agents. Antimicrob Agents Chemother.

[B26] Fichera ME, Roos DS (1997). A plastid organelle as a drug target in apicomplexan parasites. Nature.

[B27] Kohler S, Delwiche CF, Denny PW, Tilney LG, Webster P, Wilson RJ, Plamer JD, Roos DS (1977). A plastid of probable green algal origin in Apicomplexan parasites. Science.

[B28] Klepser ME, Nicolau DP, Quintiliani R, Nightingale CH (1997). Bacterial activity of low-dose clindamycin administered at 8- and 12-hour intervals against *Straphyllococcus aureus, Streptococcus pneumoniae *and *Bacteroides fragilis*. Antimicrob Agents Chemother.

[B29] Gatti G, Malena M, Caszza R, Borin M, Bassetti M, Cruciani M (1996). Penetration of clindamycin and its metabolite N-demethylclindamycin into cerebrospinal fluid following intravenous infusion of clindamycin phosphate in patients with AIDS. Antimicrob Agents Chemother.

[B30] Flaherty JF, Rodondi LC, Guglielmo BJ, Fleishaker JC, Townsend BJ, Gambertoglio JG (1998). Comparative pharmacokinetics and serum inhibitory activity of clindamycin in different dosing regimens. Antimicrob Agents Chemother.

